# Elucidating Solution-State Coordination Modes of Multidentate Neutral Amine Ligands with Group-1 Metal Cations: Variable-Temperature NMR Studies

**DOI:** 10.1021/acs.inorgchem.2c02457

**Published:** 2022-09-16

**Authors:** Nathan Davison, James A. Quirk, Corinne Wills, Casey Dixon, Paul G. Waddell, James A. Dawson, Erli Lu

**Affiliations:** Chemistry-School of Natural and Environmental Sciences, Newcastle University, Newcastle upon Tyne NE1 7RU, U.K.

## Abstract

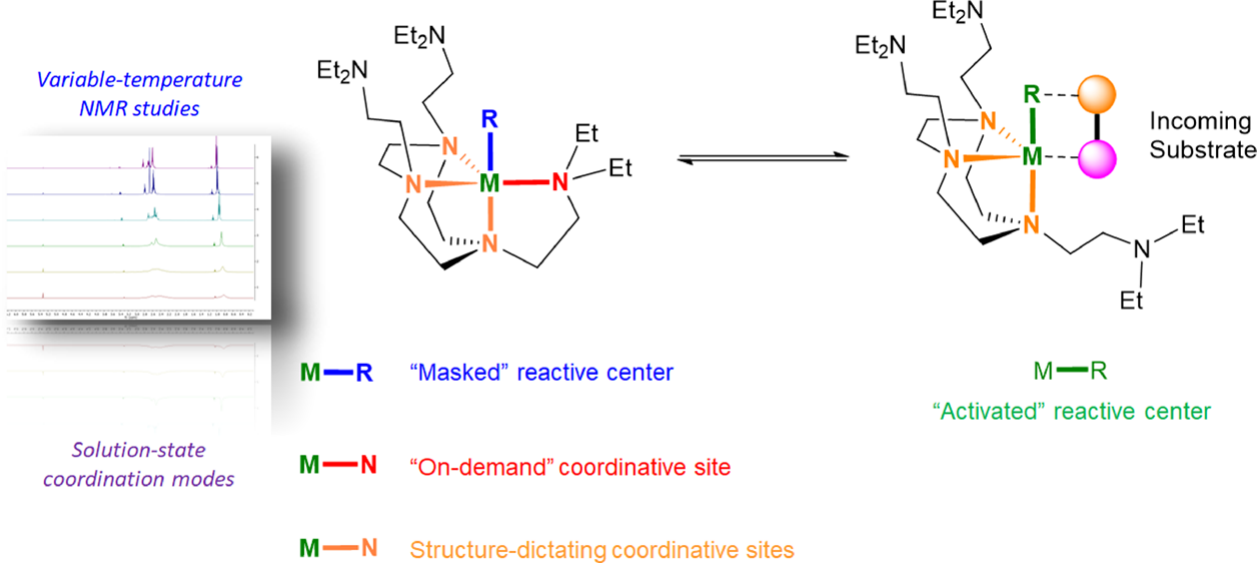

Multidentate neutral amine ligands play vital roles in coordination chemistry and catalysis. In particular, these ligands are used to tune the reactivity of Group-1 metal reagents, such as organolithium reagents. Most, if not all, of these Group-1 metal reagent-mediated reactions occur in solution. However, the solution-state coordination behaviors of these ligands with Group-1 metal cations are poorly understood, compared to the plethora of solid-state structural studies based on single-crystal X-ray diffraction (SCXRD) studies. In this work, we comprehensively mapped out the coordination modes with Group-1 metal cations for three multidentate neutral amine ligands: tridentate 1,4,7-trimethyl-1,4,7-triazacyclononane (Me^3^TACN), tetradentate tris[2-(dimethylamino)ethyl]amine (Me^6^Tren), and hexadentate N,N′,N″-tris-(2-N-diethylaminoethyl)-1,4,7-triaza-cyclononane (DETAN). The macrocycles in the Me^3^TACN and DETAN are identified as the rigid structural directing motif, with the sidearms of DETAN providing flexible “on-demand” coordination sites. In comparison, the Me^6^Tren ligand features more robust coordination, with the sidearms less likely to undergo the decoordinating–coordinating equilibrium. This work will provide a guidance for coordination chemists in applying these three ligands, in particular, the new DETAN ligand to design metal complexes which suit their purposes.

## Abstract 2

Combining variable-temperature nuclear magnetic resonance (VT NMR) and DFT calculations, this work elucidates the solution-state coordination modes of three multidentate neutral amine ligands with Group-1 metal cations. Our studies prove that two ligand-design building blocks, that is, N-macrocycle (TACN) and N-sidearms, act as structure-dictating and hemilabile coordinating sites, respectively. The concept could be utilized in designing new catalytic systems, which anchor the metal center on the macrocycle, while the sidearms serve as “on-demand” protective coordination sites.

## Introduction

Ligands play vital roles in coordination chemistry and catalysis. They provide the platform to tune the structures of metal complexes and therefore to modify their reactivity. Hence, understanding the coordination modes of ligands toward the metal center is the central topic of coordination chemistry. The primary method to obtain the knowledge of coordination modes is single-crystal X-ray diffraction (SCXRD), which can provide precise structural information including coordination geometries, bond lengths/angles, and so on. However, though SCXRD is a powerful tool (if not the most powerful one), it has its inherent limits. For example, SCXRD can only provide solid-state structural information, which does not necessarily reflect the metal complex structures in solution. The solution-state structure, on the other hand, is of paramount importance for understanding the reactivity of metal complexes since the majority of stoichiometric and catalytic reactions happen in solution.

Arguably, the solid-state *vs* solution-state structural discrepancy is greatest for Group-1 metal complexes.^1^ Since the Group-1 metal–ligand bond is mainly ionic, and the coordination field theory does not operate in this regime, that is, Group-1 metal complex structures are considered highly fluxional and labile to change corresponding to their surrounding environments, such as solvents. This structural lability renders the solid- vs solution-state structural discrepancy a debatable topic in Group-1 metal coordination chemistry for decades.^2^ From a practical perspective, understanding solution-state structures of Group-1 metal complexes, such as how the Group-1 metal centers interact with external ligands in solution, is, arguably, of more importance than their solid-state SCXRD structures. This is due to the fact that most, if not all, of the widely applied Group-1 metal reagents, such as the ubiquitous organolithium or lithium amide reagents, need to operate in solution. However, despite the importance, the structural characterization data of Group-1 metal complexes is highly unbalanced: a plethora of Group-1 metal complexes have been characterized by the SCXRD studies,^1,3,4^ but much fewer efforts have been made to elucidate their solution-state structures.

Specifically, multidentate neutral amine ligands are arguably the most successful ligand family in Group-1 metal coordination chemistry.^5^ These ligands feature tunable steric profiles, thermodynamically robust backbones, as well as flexible and versatile coordination modes. Therefore, they have been widely used to pursue highly reactive Group-1 metal complexes. For example, the bidentate *N,N,N’N’*-tetramethylethylenediamine (TMEDA) ligand was widely used to coordinate to common lithium reagents, such as *n*-butyllithium^6,7^ and lithium diisopropyl amide (LDA).^8^ Another bidentate diamine ligand, namely, (−)-Sparteine, was used to isolate the first monomeric *tert*-butyllithium complexes.^9,10^ Tetradentate *tris*-[2-(dimethylamino)ethyl]amine (Me^6^TREN) was employed to synthesize a series of Group-1 metal benzyl complexes^11^ and a (trimethylsilyl)methyllithium monomeric complex^12^ and was applied in Group-1 metal catalysis.^13^ Recently, we reported a new *hexa*-dentate amine ligand, namely, *N*,*N*′,*N″*-*tris*-(2-*N*-diethylaminoethyl)-1,4,7-triaza-cyclononane (DETAN), which enabled the isolation and characterization of the first monomeric complex of the parent organolithium reagent: methyllithium.^14^ Solid-state SCXRD studies revealed that the DETAN ligand features versatile coordination modes with Group-1 metal cations depending on the metal ionic radii and metal substituents.^15^ We would like to bring to our readers' awareness that, other than the multidentate ligands (such as the DETAN), another strategy to isolate MeLi from its aggregates is via multimetallic chelating, such as in a Li_3_(μ-Me) complex [(thf)_3_Li_3_(μ-Me){(N^*t*^Bu)_3_S}] reported by the Stalke group in 2001,^16^ and a Mo(μ-H)Li(μ-Me)Mo complex reported by the Carmona group in 2022.^17^ In these two complexes, the anionic methyl group (CH_3_^–^), though in its monomeric form, occupies a bridging position and shared by multiple metal sites.

Most of these aforementioned structural studies are based on solid-state SCXRD structures. Solution-state structural studies of Group-1 metal complexes have been far less explored.^18^ The degree of aggregation of organolithium complexes (such as *n*-butyl lithium) in solution is a crucial topic in organolithium-mediated reactions. The Stalke group and co-workers have thoroughly investigated the organolithium aggregations employing a variety of NMR techniques, such as ^7^Li and ^1^H diffusion-ordered spectroscopy (DOSY),^19,20^ exchange spectroscopy (EXSY),^21^ nuclear Overhauser effect (NOE),^22^ residual quadrupolar couplings,^23^ and variable-temperature (VT) NMR.^24^ The Collum group also used NMR methods to elucidate Group-1 metal alkyl/amide complexes’ reactivity, kinetics, and reaction mechanisms in solution.^8,25−27^ These NMR works mainly focused on *intermolecular* processes, such as aggregating-deaggregating equilibrium, intermolecular reactions with incoming substrates, and elucidating cluster sizes. On the other hand, the solution-state *intramolecular* processes of Group-1 metal complexes, such as the ligand geometry fluxion and coordination–decoordination equilibrium of ligand fragments (not the whole ligand but parts of the ligand), are far less explored. To the best of our knowledge, the only precedent of such study was conducted by the Stalke group in 2014, where they employed a combined SCXRD, NMR, and computational approach to study the intramolecular coordination changes of a multicomponent lithium lithiate.^28^ These intramolecular solution-state dynamic processes have significant influences over the reactivity of the Group-1 metal complexes; therefore, addressing these processes is not only of interest to the coordination chemistry community but will also benefit the organic and catalysis communities.

**Figure 1:**
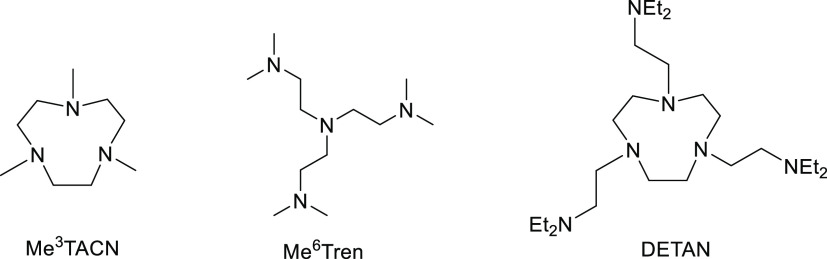
Three ligands of topic in this work.

In this work, employing VT-NMR as the method, we report comprehensive studies of solution-state ligand coordination modes of three multidentate amine ligands with Group-1 metal cations Li^+^ and Na^+^. The three ligands studied in this work are (1) *tris*-dentate 1,4,7-trimethyl-1,4,7-triazacyclononane (Me^3^TACN); (2) *tetra*-dentate Me^6^Tren; and (3) *hexa*-dentate DETAN (Figure 1). This work unveils different coordination behaviors of two different ligand fragments (the macrocycle and the sidearm), which can guide chemists to choose/design suitable ligands in their future work. The details of our findings are elaborated in the following sections.

## Results and Discussion

**Figure 2:**
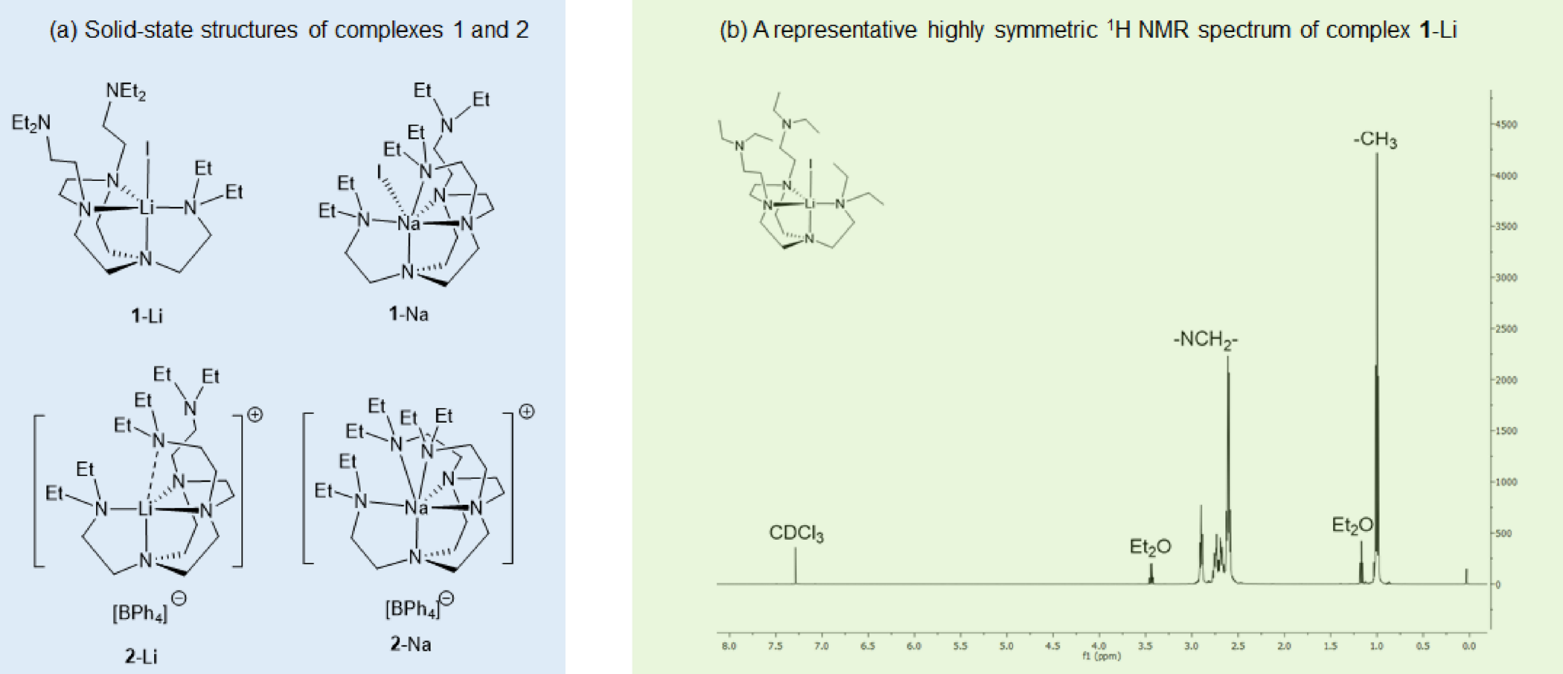
(a) Solid-state structures of complexes **1** and **2**. (b) Representative ^1^H NMR spectrum: complex **1**-Li in CDCl_3_ at 298 K [15].

During our studies of the DETAN-coordinated lithium and sodium complexes **1** and **2** (Figure 2),^15^ we noticed apparent discrepancies between their solid-state SCXRD structures and room-temperature NMR spectra. For instance, the solid-state structure of complex **1**-Li features a *pseudo*-*C*_s_ symmetry: one of the three ligand sidearms coordinates to the metal center, while the other two remain uncoordinated (Figure 2a). If this *pseudo-C*_s_-symmetric solid-state structure retains in solution, there should be two groups of sidearm signals with a ^1^H integral ratio of 2:1: one group represents the coordinated sidearm and the other group represents the two uncoordinated sidearms. However, the ^1^H NMR spectrum of complex **1**-Li (Figure 2b) at 298 K exhibits a higher-than-expected symmetry: there is only one set of the sidearm signals (the diagnostic NMR probe is the triplet of NCH_2_C*H*_3_ at approximately 1.0 ppm), indicating a *pseudo*-*C*_3_*v* symmetry at this temperature (298 K) at the NMR timescale. Similar highly symmetric NMR spectra were observed for complexes **1**-Na^29^ and **2**-Li/Na, despite their diversified solid-state structures with lower symmetries.

We hypothesize that these apparent discrepancies between the solid-state structures and the solution-state ^1^H NMR spectra of complexes **1** and **2** originate from the DETAN ligand’s fluxional coordinating behaviors. In complexes **1** and **2**, the DETAN ligand coordinates to the metal centers via dative N → M bonds, which are labile to coordination–decoordination equilibrium. To further understand the fluxional coordinating behaviors, we conducted VT ^1^H NMR studies of complexes **1** and **2**.

**Figure 3:**
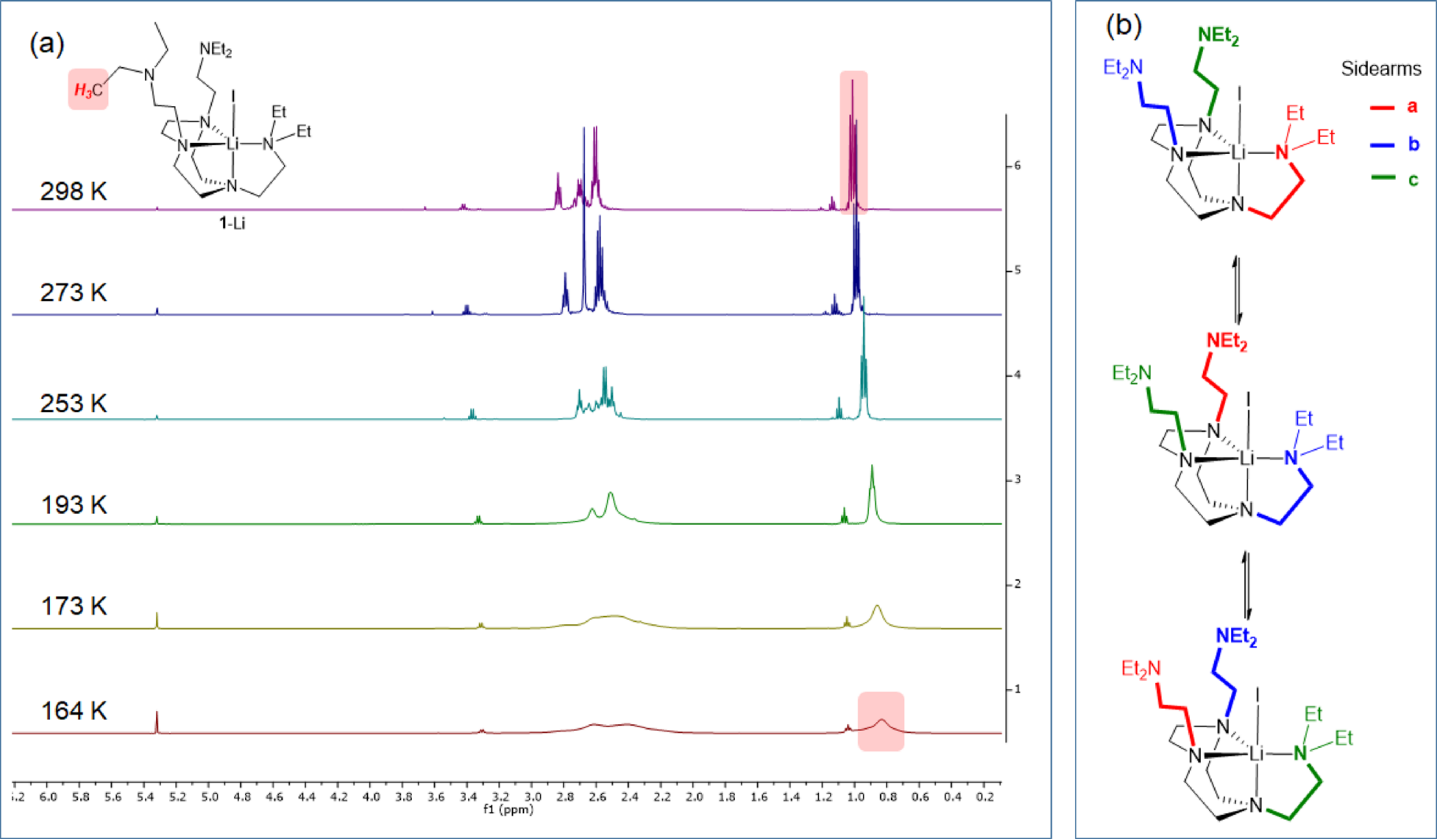
(a) VT ^1^H NMR spectra of **1**-Li in CD_2_Cl_2_. (b) Schematic presentation of the coordination–decoordination equilibrium. The three sidearms are differentiated through their color codes.

We first focus on the ^1^H NMR signals of–NCH_2_C*H*_3_ of the DETAN sidearms since the −CH_3_ chemical environment is diagnostic for the coordination status of the sidearms. The VT ^1^H NMR spectra of **1**-Li are exhibited in Figure 3a. Upon cooling from 298 to 164 K, the sidearm −C*H*_3_ signal of **1**-Li broadens but does not decoalesce into multiple signals. We hypothesize that the three sidearms in **1**-Li undergo a fast coordination–decoordination equilibrium, as illustrated in Figure 3b. The broadening of the −C*H*_3_ signal at lower temperatures suggests that the exchange is slowing down but is still relatively rapid at the NMR timescale. We have not been able to slow down the exchange sufficiently to see two distinctive sets of sidearm signals as is suggested by the SCXRD structure, that is, even 164 K is not cold enough to “freeze” the sidearms’ fluxional behavior. Temperatures lower than 164 K are not available due to the melting point of the NMR solvent *d*_2_-dichloromethane (CD_2_Cl_2_).

**Figure 4:**
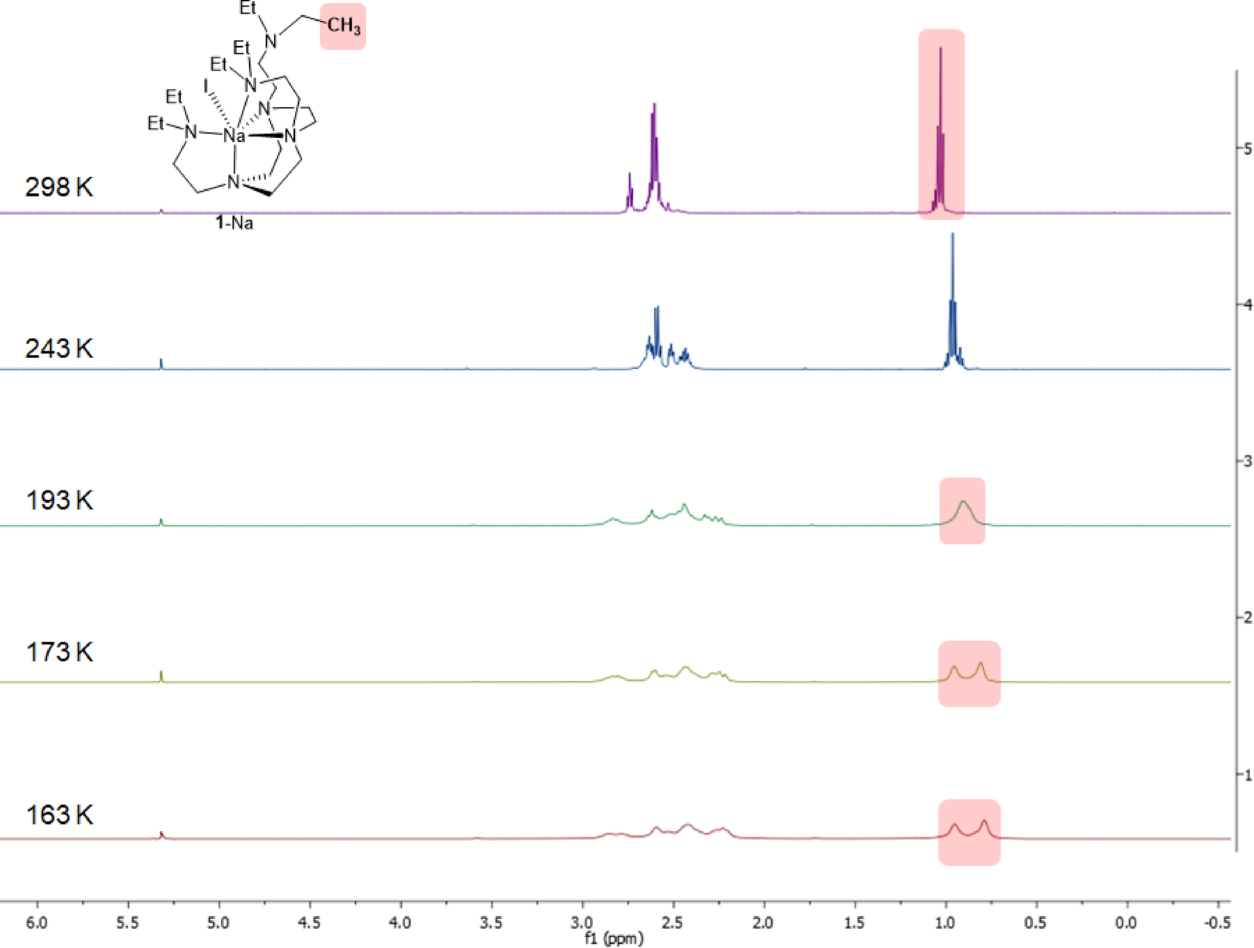
VT ^1^H NMR spectra of **1**-Na in CD_2_Cl_2_.

Other than temperature, the other factor which could influence the sidearm coordination–decoordination equilibrium is the metal identity: the N → M dative bond strengths are different with different metals (M); therefore, their coordination–decoordination equilibrium constants will be different. We hypothesized that changing from Li^+^ to Na^+^ could render the splitting of the sidearm −C*H*_3_ signals observable within the available temperature range. Indeed, to our gratification, the VT ^1^H NMR spectra of **1**-Na exhibit two sets of −C*H*_3_ signals upon cooling to 173 K (Figure 4), which are of an integral ratio of 2:1, reflecting the SCXRD structure of complex **1**-Na.

**Figure 5:**
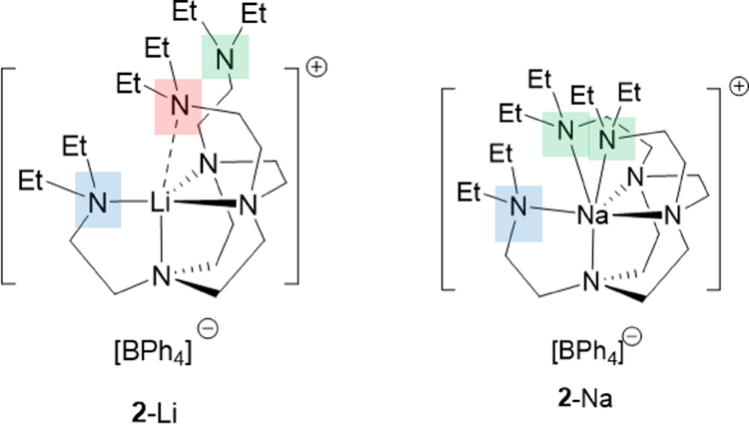
Schematic representations of the SCXRD structures of **2**-Li/Na. The sidearms are grouped and color-coded according to their chemical environments.

Other than the halide complexes **1**-Li/Na, similar sidearm coordination–decoordination equilibrium can be observed in the separated ion pair (SIP) complexes **2**-Li/Na. Considering their SCXRD structures, the situations in the SIP complexes **2**-Li/Na are more complicated. In **2**-Li, the three sidearms are each in different chemical environments (Figure 5), rendering the [Li^+^(DETAN)] cation chiral in its solid state. For Na^+^, which has the larger ionic radius, the three sidearms divide into two groups, with significantly different N → Na bond lengths (∼2.90 Å vs ∼2.75 Å); therefore, the [Na^+^(DETAN)] cation features a *pseudo*-*C*_s_ symmetry (Figure 5).

**Figure 6:**
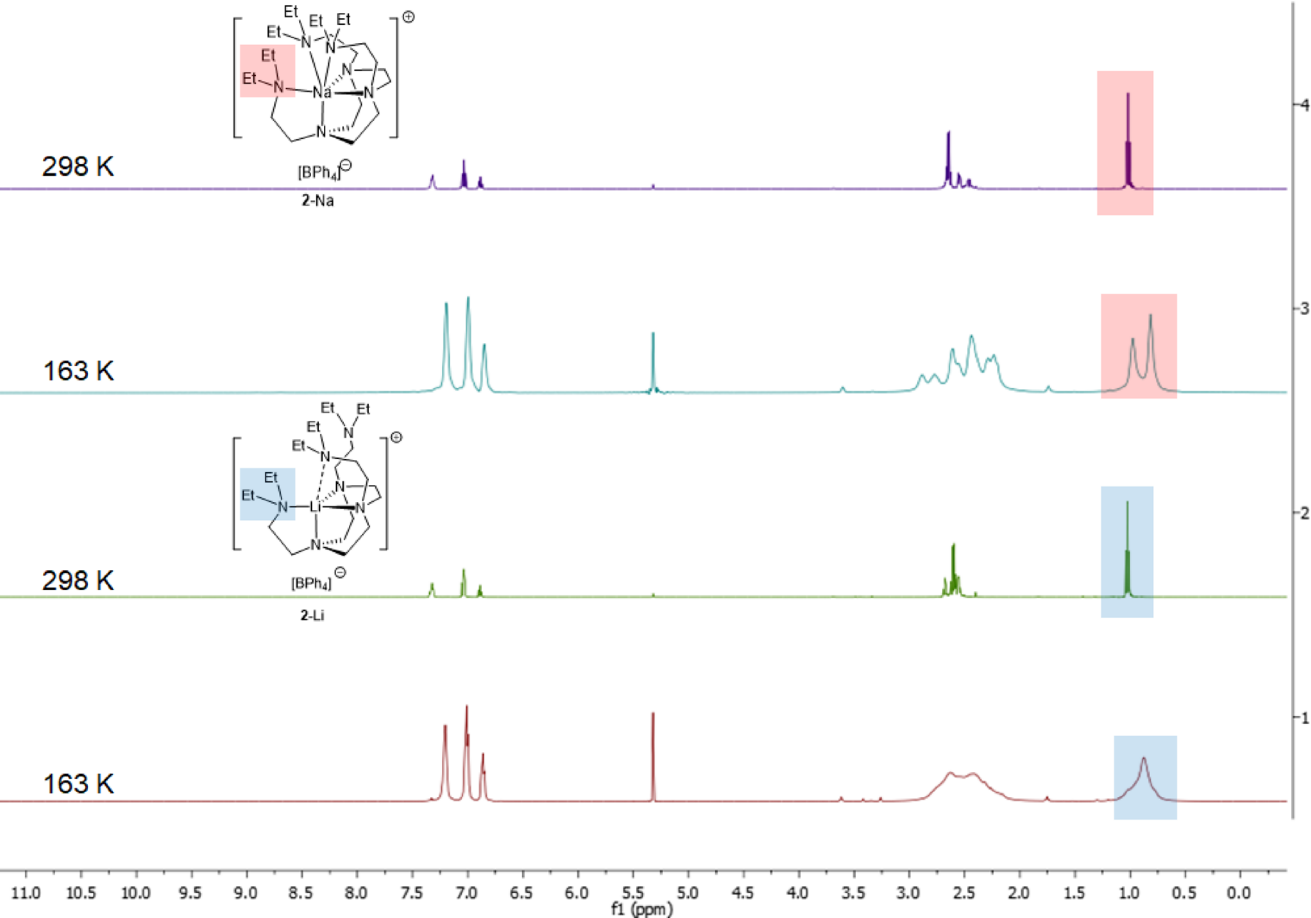
VT ^1^H NMR spectra of **2**-Li/Na in CD_2_Cl_2_. The top two spectra: ^1^H NMR spectra of **2**-Na at 298 and 163 K, respectively. The bottom two spectra: ^1^H NMR spectra of **2**-Li at 298 and 163 K, respectively.

The ^1^H NMR spectra of **2**-Li/Na in CD_2_Cl_2_, on the other hand, suggest higher symmetries at 298 K (Figure 6), similar to what we have discussed in **1**-Li/Na. Upon cooling to 163 K, the sidearm signals of **2**-Li broadened but did not decoalesce, though a small shoulder starts to appear at *circa* 1.0 ppm, which could be the second set of sidearm signals. In comparison, for **2**-Na, the sidearms split into two groups at 163 K. These observations corroborate our hypothesis that different N → M dative bond strengths have a noteworthy influence on the DETAN’s sidearm coordination–decoordination equilibrium in solution.

**Figure 7:**
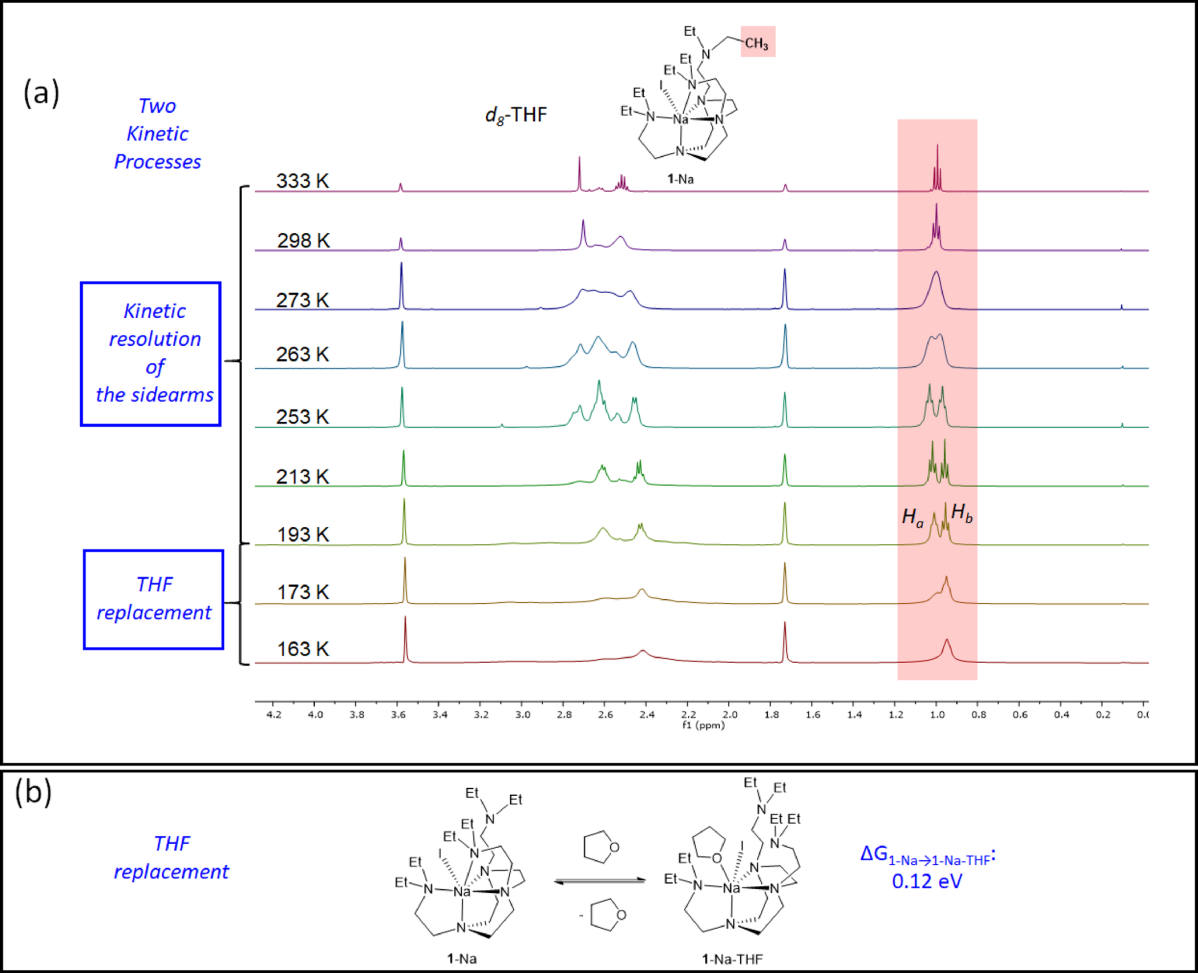
(a) VT ^1^H NMR spectra of **1**-Na in *d*_8_-THF. (b) Schematic representation of the THF replacement process and the calculated energy difference between **1**-Na and **1**-Na-THF.

The previous discussions of the kinetic behaviors of complexes **1** and **2** are based on VT ^1^H NMR studies in a *non-coordinative* solvent CD_2_Cl_2_. An intuitive and immediate question would be as follows: what is the effect of *coordinative* solvents (such as THF)? To address the question, we conducted VT ^1^H NMR studies of complexes **1** and **2** in *d*_8_-THF. For the Li^+^ complexes **1**-Li and **2**-Li, we observed similar kinetic behaviors in *d*_8_-THF and CD_2_Cl_2_ (**1**-Li: Figure S1 in *d*_8_-THF *cf.*Figure 3 in CD_2_Cl_2_; **2**-Li: Figure S2 in *d*_8_-THF *cf.*Figure 6 bottom in CD_2_Cl_2_). Therefore, we postulate that the external coordinative solvent, such as THF, plays little role in the coordinative kinetic of the Li^+^ complexes. This is a sensible postulation: the DETAN ligand could provide a saturated inner coordinative sphere for the relatively small Li^+^ cation through relatively strong N → Li dative bonds, preventing the external THF molecules from coordinating. However, for the larger ionic radius Na^+^ complex **1**-Na, its VT ^1^H NMR spectra in *d*_8_-THF (Figure 7) exhibit distinct features compared to the spectra in CD_2_Cl_2_ (Figure 4). In CD_2_Cl_2_, only one kinetic process was observed, which is the resolution of the sidearms. In comparison, in *d*_8_-THF, we observed *two* processes. From 333 to 193 K, the sidearm −C*H*_3_ signal splits from one set (333 to 263 K) into two sets (263 to 193 K), which is similar to what we have discussed in CD_2_Cl_2_, that is, a kinetic resolution of the sidearms. Intriguingly, further cooling from 213 K initially results in one of the two sets of −C*H*_3_ signal broadening (*H*_a_ at 193 K, Figure 7a), and then the two sets of signals (*H*_a_ and *H*_b_) coalesce at *circa* 163 K. *H*_a_ and *H*_b_ represent the two environments of the DETAN sidearms, which are in correspondence with **1**-Na’s solid-state structure. We hypothesize that the second kinetic process from 193 to 163 K is a THF replacement of the coordinated sidearms, resulting all the three sidearms coordination-free and hence in similar (though not identical) chemical environments, as exhibited in Figure 7a 163 K and schematically presented in Figure 7b.

The hypothetical THF replacement process is supported by DFT calculations. We conducted geometry optimizations for the structures of **1**-Na and **1**-Na-THF, respectively, at ωB97X-V level of theory (see Supporting Information for details). The optimized geometry of **1**-Na (Figure S9) replicates what we observed from its SCXRD study (Figure S7). The hypothetic **1**-Na-THF features a distorted octahedral geometry (Figure 7b; Figure S10), with one coordinated sidearm replaced by a THF molecule. It is intriguing that one of the two coordination-free sidearms in **1**-Na-THF poses its N-lone pair toward the sodium center, which could indicate some level of weak N···Na interaction. Conversion from **1**-Na to **1**-Na-THF was calculated to be slightly endothermic (0.12 eV, *circa* 2.8 kcal mol^–1^), which is in correspondence with the fact that such a THF-replacement was observed at low temperatures. Though we only calculated the *mono*-THF replacement, it is sensible to extrapolate that, with the presence of a largely excess amount of THF molecules, such as using THF as the solvent, all the DETAN sidearms could be replaced by coordinating THF molecules.

**Figure 8:**
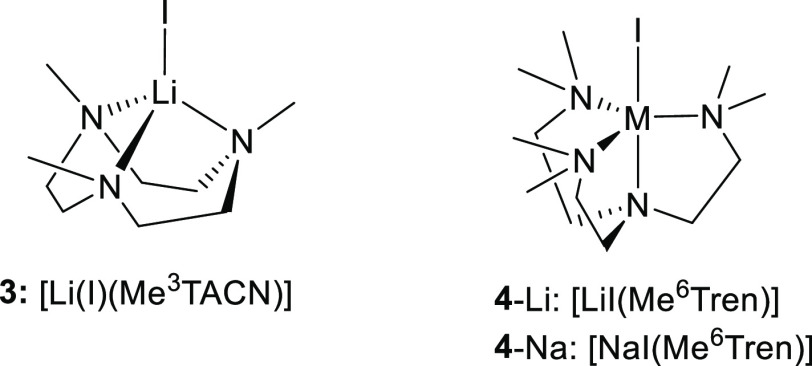
Schematic representations of the SCXRD structures of complexes **3** and **4**.

So far, our discussion has focused on DETAN’s sidearms. The other key fragment of the DETAN ligand is its TACN-based macrocycle backbone. The SCXRD solid-state structures^15^ of **1**-Li/Na and **2**-Li/Na suggested that the N → M dative bonds in the macrocycles are much shorter compared to those in the sidearms (Δ_N⃗M_: ∼0.4 Å), indicating that the macrocycle is the structural dictating group, while the sidearms act as weakly bonded hemilabile pendant groups. To prove this hypothesis, the coordination behaviors of the macrocycle and the sidearms must be disentangled and studied separately. However, this task is difficult in the DETAN complexes **1** and **2** for two reasons: (1) the ^1^H signals of the sidearms and the macrocycle overlap and (2) the macrocycle and sidearms may have complex interplay in complexes **1** and **2**, which will complicate the discussion. To disentangle the macrocycle and sidearms, we employed three model complexes, namely, [Li(I)(Me^3^TACN)] (**3**) and [M(I)(Me^6^Tren)] (**4**-Li/Na), which feature only the macrocycle (**3**) or the sidearms (**4**). The synthesis and characterization of the new complex **3**, including its SCXRD structure (Figure S8), can be found in the Supporting Information. Complex **4** was reported in our previous work.^15^ The solid-state SCXRD structures of **3** and **4**(15) feature *pseudo*-*C*_3*v*_ symmetries (Figure 8).

**Figure 9:**
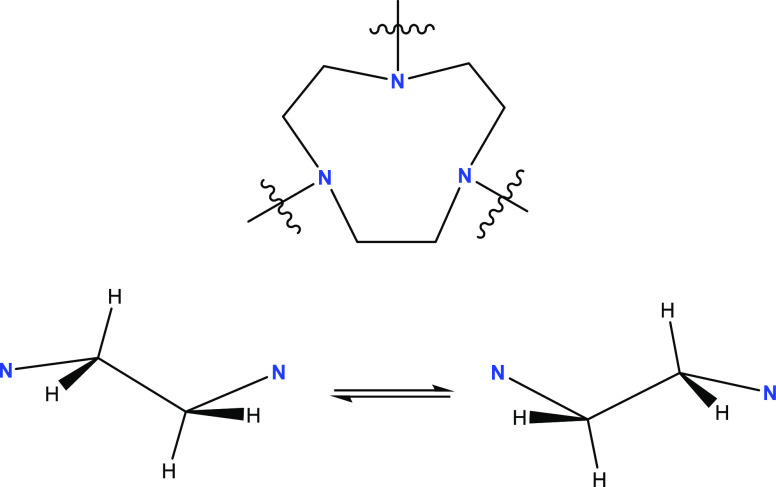
“Seesaw” conformation equilibrium of the methylene groups in the macrocycle of the Me^3^TACN ligand in complex **3**.

The VT ^1^H NMR spectra of **3** in CD_2_Cl_2_ exhibit *pseudo*-*C*_3_ symmetry at both 298 and 163 K, which is in accordance with its SCXRD structure. Hence, we conclude that, in **3**, there is no coordination–decoordination equilibrium of the macrocyclic N atoms with the Li^+^ center, that is, the TACN macrocycle coordinates strongly with the Li^+^ center. This is in line with the SCXRD structures of **3** (Figure S8) and **2**, in which the short N^TACN^ → M dative bonds between the macrocyclic N^TACN^ atoms and metal centers indicate strong interactions. A closer examination of **3**’s VT ^1^H NMR, in particular the macrocyclic methylene group (N-C*H*_2_-C*H*_2_-N) signals, suggests that these signals convert from a vicinal AA’BB’ system at 298 K to an ABCD system at 162 K (Figure S6), indicating the presence of a “seesaw” equilibrium elucidated in Figure 9. Similar robust coordination of the TACN macrocycle can be observed for the DETAN ligand in complexes **1** and **2**, as well as the “seesaw” dynamic behavior of the macrocyclic methylene signals.

**Figure 10:**
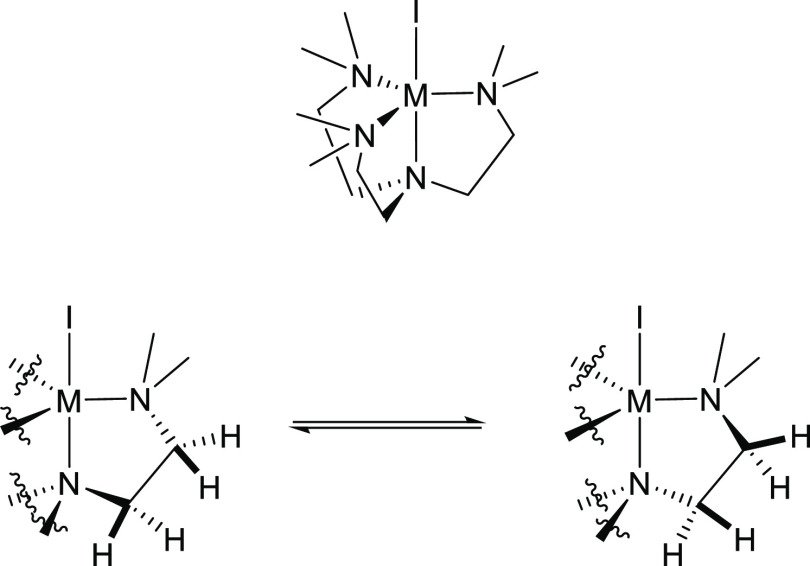
“Seesaw” conformation equilibrium of the methylene groups of the Me^6^Tren ligand in complexes **4**-Li/Na.

The VT ^1^H NMR spectra of the Me^6^Tren complexes **4**-Li/Na exhibit robust *pseudo*-*C*_3_ coordination modes within the temperature range we examined (298–178 K). The signals of the sidearm -N*Me*_2_ groups do not decoalesce at 178 K, indicating that even at this lower temperature, the three sidearms of **4**-Li/Na are still chemically equivalent. The methylene groups (NC*H*_2_C*H*_2_N) of the sidearms decoalesce from a germinal AA’BB’ spin system at 298 K to an ABCD system at 178 K, thus exhibiting the “seesaw”-type equilibrium (Figure 10), similar to the behaviors of the macrocyclic methylene groups in complex **3** (Figure 9).

We attribute the robust sidearm coordination of the Me^6^Tren ligand in complexes **4**-Li/Na to the small metal substituent, namely, iodide (I^–^). Indeed, as reported by us^12^ and others,^11,30^ the Me^6^Tren sidearms’ coordination behaviors could depend on the metal substituents. It is plausible that the relatively small iodide (I^–^) metal substituent in **4**-Li/Na allows all the three sidearms of the Me^6^Tren ligand to coordinate to the metal center. When a bulkier metal substituent, such as trimethylsilyl methyl (-CH_2_SiMe_3_), is involved, one sidearm was observed uncoordinated in our reported [Li(CH_2_SiMe_3_)(Me^6^Tren)] complex.^12^ In solution, on the other hand, a coordination–decoordination equilibrium was also observed,^12^ indicated by changing from one set of the sidearm ^1^H NMR signals at room temperature, into two sets upon cooling, which is similar to what we observed for complex **1**.

## Conclusions and Outlook

**Figure 11:**
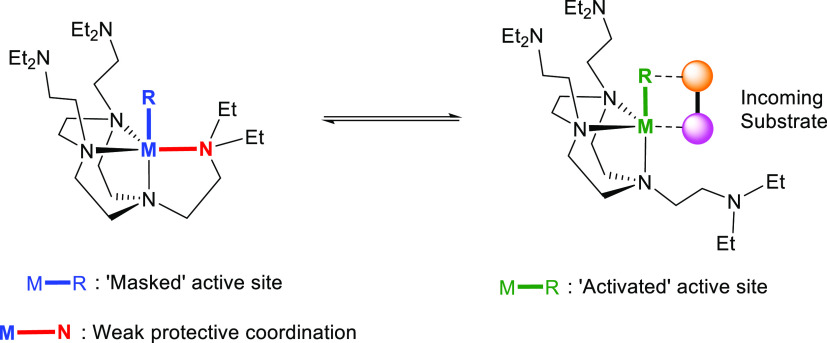
Postulated DETAN sidearm “masked” active site and its activation upon exposure to substrate(s).

This work comprehensively surveyed the solution-state coordination modes of three neutral multidentate amine ligands (DETAN, Me^3^TACN, and Me^6^Tren) with Group-1 metal cations. In complement with our previous SCXRD studies of the DETAN ligand-supported Group-1 metal complexes,^15^ this work provides a holistic understanding of the novel DETAN ligand’s coordination behaviors in both solid state and solution. Specifically, the TACN macrocycle of the DETAN ligand serves as the structural dictating motif, which is responsible for metal ionic radii selectivity. The DETAN’s sidearms, on the other hand, act as hemilabile flexible coordination sites, to provide auxiliary chelating donors, which could be essential to stabilize highly reactive species. The sidearms’ coordination is much weaker compared to the TACN macrocycle and is largely reversible, revealed by our VT ^1^H NMR studies and DFT calculations. This feature of the DETAN ligand opens an avenue toward “on-demand” catalysis: the sidearms coordinate and protect the highly reactive metal center, which could easily decoordinate to allow substrate(s) to enter the inner coordination sphere and react at the metal center (Figure 11). More work is underway to exploit this feature of the DETAN ligand to deliver unprecedented stoichiometric and catalytic reactions.

## Supporting Information Available

NMR data collection and analysis as well as the structure of **1**-Na and synthesis and characterization of complex **3**. See DOI: TBC (PDF)

## Dedication

This article is dedicated to our colleague and friend, Emeritus Professor William Clegg (Newcastle University), for his contribution to SCXRD teaching and research, and to celebrate his Max Perutz Prize of the European Crystallographic Association in 2022.
