# Supplementary material for: Elucidating Solution-State Coordination Modes of Multidentate Neutral Amine Ligands with Group-1 Metal Cations: Variable-Temperature NMR Studies
Source: Inorg Chem. 2022 Sep 16;61(38):15204–12. doi: 10.1021/acs.inorgchem.2c02457 (PMC9516690; doi:10.1021/acs.inorgchem.2c02457)
Supplement: Supplementary file 1 — ic2c02457_si_001.pdf [file ic2c02457_si_001.pdf]

Supporting Information

Elucidating Solution-State Coordination Modes of Multidentate Neutral Amine Ligands with Group-1 Metal Cations: Variable-Temperature NMR studies

Nathan Davison, James A. Quirk, Corinne Wills *, Casey Dixon, Paul G. Waddell, James A. Dawson *, Erli Lu (陆 而立) *

Chemistry-School of Natural and Environmental Sciences, Newcastle University. Newcastle upon Tyne, UK. NE1 7RU

Corresponding Authors

Erli Lu (erli.lu@newcastle.ac.uk); Corinne Wills (corinne.wills@newcastle.ac.uk); James A. Dawson (james.dawson@newcastle.ac.uk)

Contents

1. General……………………………………………………………………………...S3

2. VT ^{1}H NMR spectra of **1**-Li and **2**-Li……………………………………..………...S4

3. Synthesis and characterization of [Li(I)(Me^{3}TACN)] (**3**)…......……. ……………S5-S9

4. Single crystal X-ray diffraction studies of **1**-Na and **3**……………………………S9-S12

5. Computational details and calculated structures of 1-Na and 1-Na-THF………S13-S14

6. References.....……………………………………………………….. ……………S15

General procedures

All manipulations were carried out using Schlenk techniques, or in a Vigor glovebox under a dry argon atmosphere. Aliphatic, aromatic and ethereal solvents were dried by sodium metal and sodium/potassium alloy, then distilled under vacuum. Halogenated solvents were dried over activated 4 Å molecular sieves, degassed by three freeze-pump-thaw cycles and stored under argon. *Tris*[2-(dimethylamino)ethyl]amine (Me_6Tren) and 1,4,7-Trimethyl-1,4,7-triazacyclononan (Me_3TACN) were purchased from Merck and dried over 4 Å molecular sieves prior to use. *N,N',N"-Tris*-(2-N-diethylaminoethyl)-1,4,7-triaza-cyclononane (DETAN) was prepared as previously reported [1]. Complexes **1**, **2** and **4** were prepared following our reported procedures [2]. LiI was purchased from Merck and dried under dynamic vacuum for 12 hours and stored in the glovebox prior to use.

1H, $^{13}C\{^{1}H\}$ and ^{7}Li NMR spectra were recorded on a Bruker 300 Avance III spectrometer operating at 300.13, 75.48 and 116.64 MHz respectively. Chemical shifts are quoted in ppm and are relative to $SiMe_4$ (^{1}H and ^{13}C) or external 0.1M LiCl in D_2O (^{7}Li). Variable temperature ^{1}H NMR experiments were carried out on a Bruker Avance III HD spectrometer operating at 500.15 MHz.

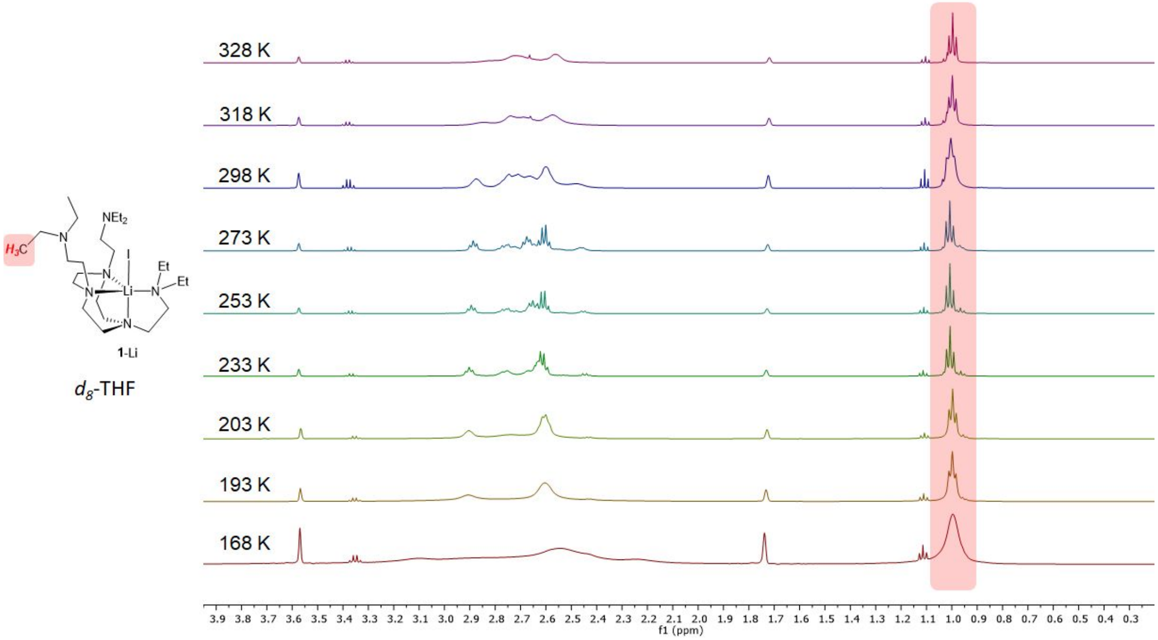

Figure S1. VT ^{1}H NMR spectra of **1**-Li in d_8-THF.

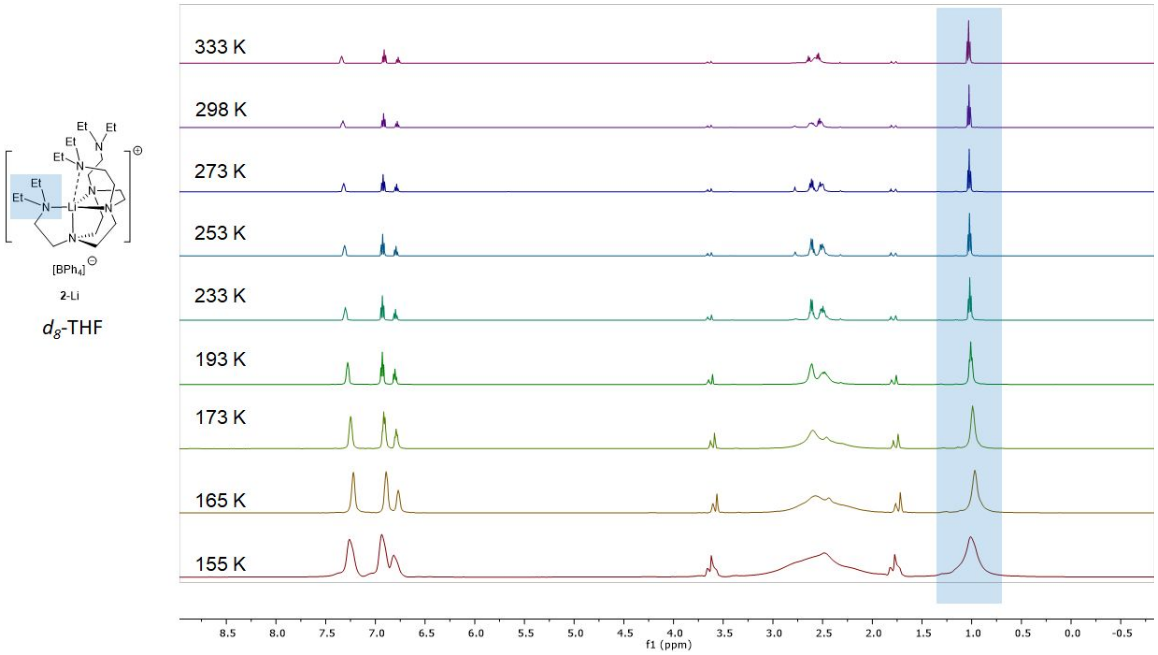

Figure S2. VT ^{1}H NMR spectra of **2**-Li in d_8-THF.

Synthesis of [Li(I)(*κ*^{3}-*N,N'N"*-Me^{3}TACN)] (3)

Me_3TACN (0.514 g, 3 mmol) and LiI (0.402 g, 3 mmol) were combined in a 50 ml ampoule. Et_2O (20 ml) was added at room temperature and the resulting mixture was allowed to stir for 18 hours. The mixture was filtered, and the resulting white solid was washed with hexane (20 ml) and dried *in vacuo* to afford **3** as a white solid (74% yield). Colourless crystals suitable for SCXRD were obtained by dissolving 3 (0.250 g) in boiling THF (20 ml) and cooling the resulting solution to 4 ^{o}C.

^{1}H NMR (700 MHz, CD_2Cl_2, 25 °C): δ (ppm) 2.65 – 2.60 (m, 6H, NCH_2CH_2N), 2.56 – 2.51 (m, 6H, NCH_2CH_2N), 2.52 (s, 9H, NCH_3)

^{13}C NMR (75 MHz, $CDCl_3$, 25 °C): δ (ppm) 53.4 (NCH$_2$$CH_2$N), 46.3 (N$CH_3$)$_2$)

^{7}Li NMR (117 MHz, $CDCl_3$, 25 °C): δ (ppm) 3.24

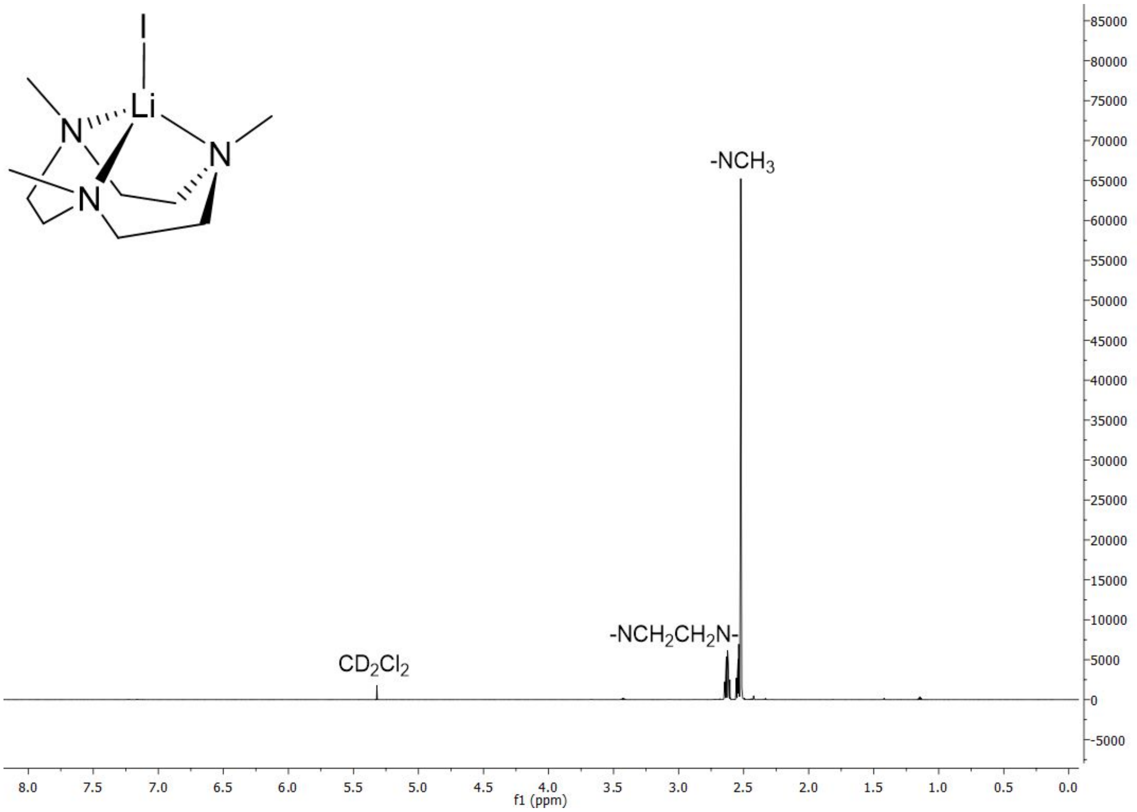

Figure S3. ^{1}H NMR spectrum of complex **3** in CD_2Cl_2 at 298 K.

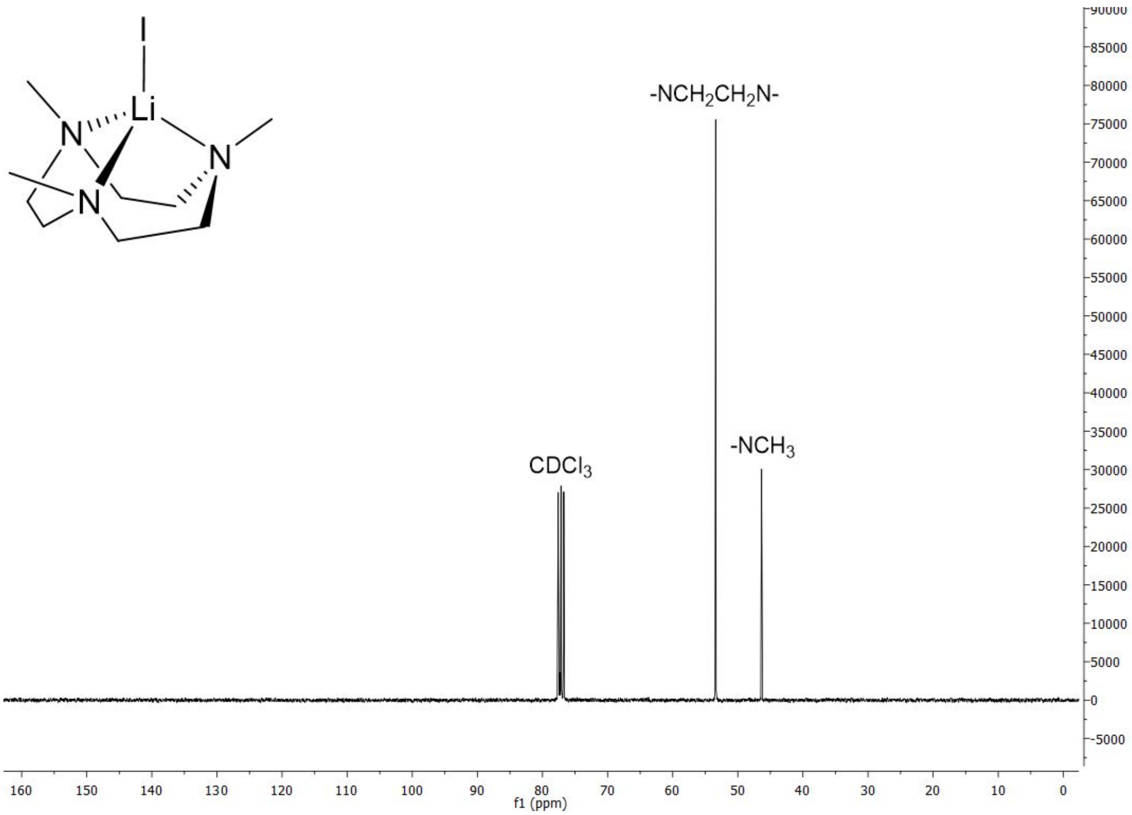

Figure S4. ^{13}C NMR spectrum of complex **3** in $CDCl_3$ at 298 K.

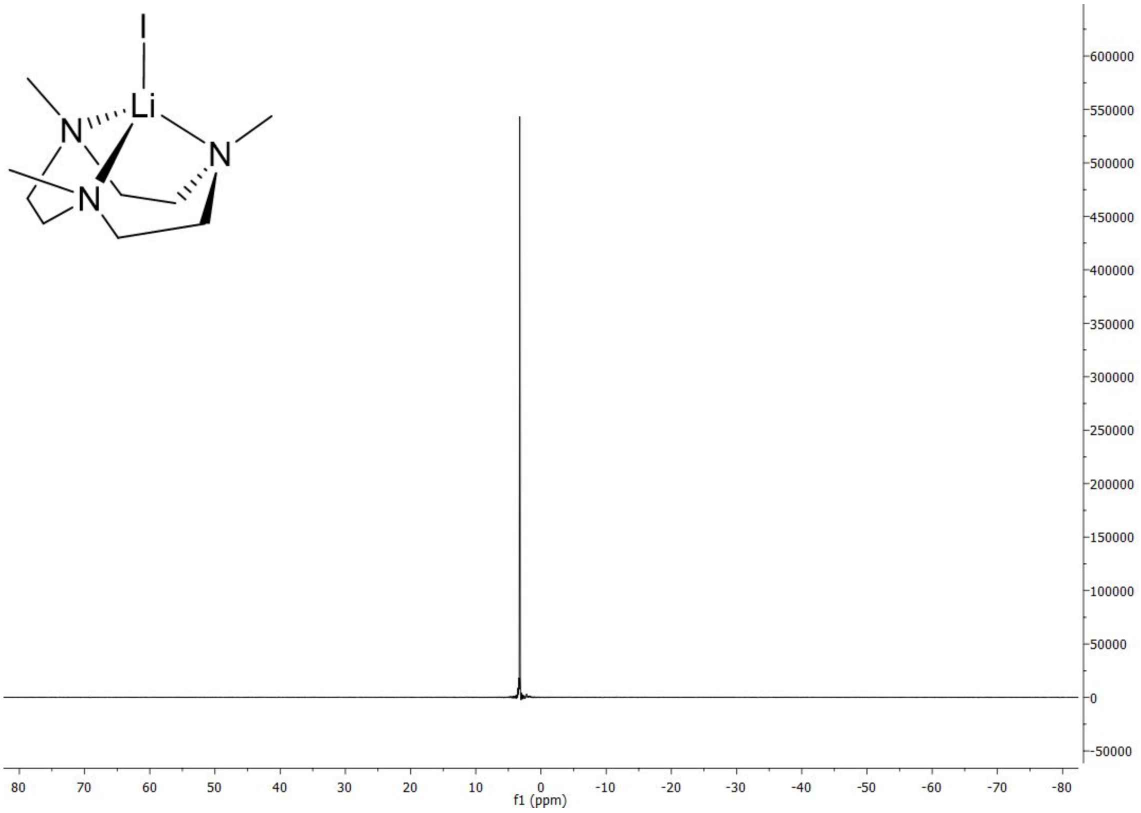

Figure S5. ^{7}Li NMR spectrum of complex **3** in $CDCl_3$ at 298 K.

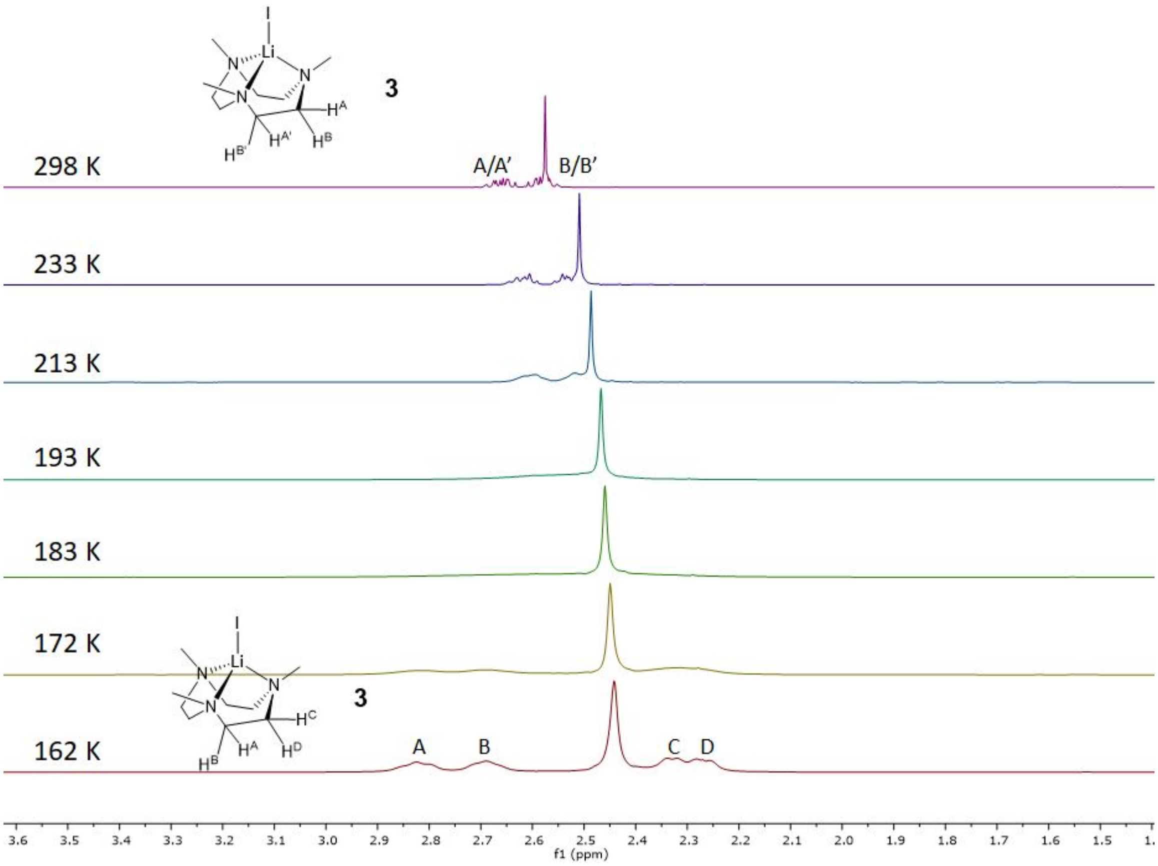

Figure S6. Variable temperature ^{1}H NMR spectra of complex **3** in CD_2Cl_2 to exhibit the change of the methylene protons from the AA'BB' system at 298 K to the ABCD system at 162 K.

Single-crystal X-ray diffraction (SCXRD) details

Data for the structures of **3** were collected 150 K on a Rigaku Oxford Diffraction Xcalibur, Atlas, Gemini ultra diffractometer equipped with an Oxford Cryosystems CryostreamPlus open-flow N_2 cooling device using copper radiation ($\lambda_{CuK\alpha}$ = 1.54184 Å). The intensities were corrected for absorption empirically using spherical harmonics. Cell refinement, data collection and data reduction were undertaken via the software CrysAlisPro[3]; solved using XT[4] and refined by XL[5] using the Olex2 interface[6]. All non-hydrogen atoms

were refined anisotropically and hydrogen atoms were positioned with idealised geometry and their atomic displacement parameters (ADP) constrained to be an appropriate multiple of the parent atom.

Table S1. Crystal Structure Refinement Details for Complexes

Complex	**1**-Na	**3**
Empirical formula	C24 H54 I N6 Na	C9 H21 I Li N3
Formula weight	576.62	305.13
Temperature/K	150 K	150 K
Crystal system	orthorhombic	Monoclinic
Space group	Pbcn	P 21/n
a/Å	20.5799(5)	12.0377(5)
b/Å	20.3489(4)	8.7164(3)
c/Å	14.2880(3)	12.9224(4)
α/°	90	90
β/°	90	93.726(3)
γ/°	90	90
Volume/Å^3	2983.5(2)	1353.02(8)
Z	8	4
ρ_{calc}g/cm^3	1.280	1.498
μ/mm^{-1}	8.700	4.002
F(000)	2432.0	608.0
Crystal dimensions (mm)	0.5 x 0.14 x 0.05	0.33 x 0.15 x 0.06
Crystal colour	Colourless	Colourless
Radiation	Cu Kα	Cu Kα
2Θ range for data collection/°	4.297 to 66.513	4.866 to 66.674
Goodness-of-fit on F^2	1.043	1.1485
Reflections, parameters, restraints	5265, 356, 563	4278, 132, 0

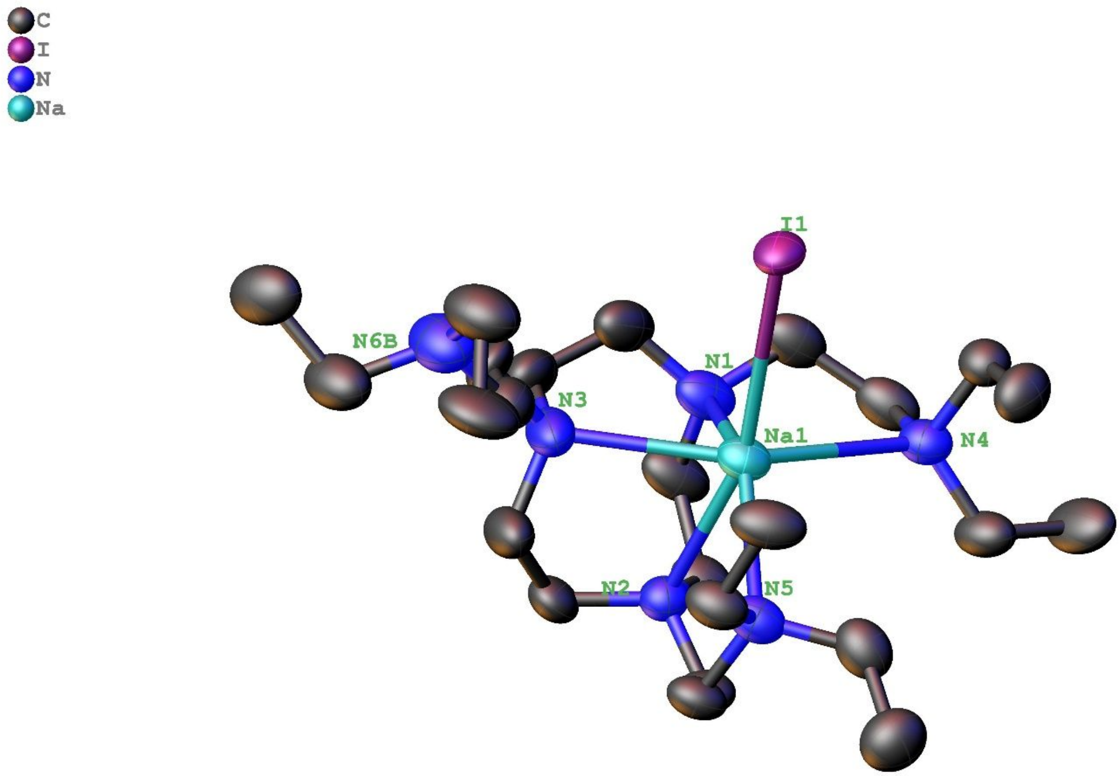

Figure S7. Molecular structure of **1**-Na. Selected bond lengths (Å): Na1-I1 3.155(2); Na1-N1 2.430(6); Na1-N2 2.514(5); Na1-N3 2.614(6); Na1-N4 2.856(6); Na1-N5 2.604(6).

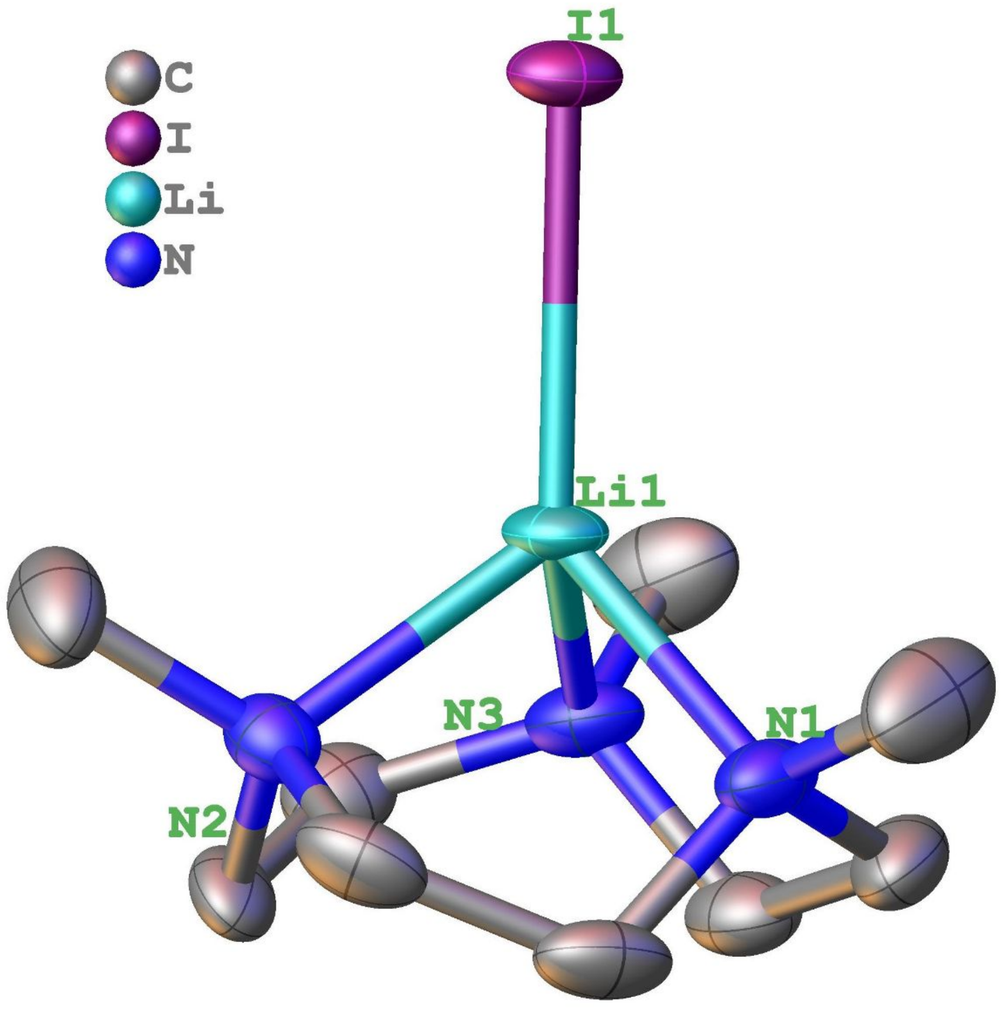

Figure S8. Molecular structure of **3**. Key bond lengths (Å): Li1−I1 2.624(11), Li1−N1 2.065(14), Li1−N2 2.055(14), Li1−N3 2.066(13).

Computational details

General: Density functional theory calculations were carried out in CP2K[7] with the ωB97X-V hybrid functional.[8] The geometry of each structure (**1**-Na, THF, and **1**-Na-THF) was optimized until the forces on atoms were less than 0.01 $eVÅ^{-1}$. Simulations were performed using the wavelet solver in a non-periodic cubic cell with lengths of 25 Å. We use triple-ζ basis sets optimized from molecular calculations (MOLOPT) [9] and the Goedecker-Teter-Hutter pseudopotentials available within CP2K.[10],[11],[12] We use four multigrids with a relative cutoff of 50 Ry and the finest grid having a cutoff of 1200 Ry. The computational cost of hybrid calculations is reduced using the auxiliary density matrix method [13],[14] in which exchange integrals are approximated through mapping onto smaller, more localized basis sets.

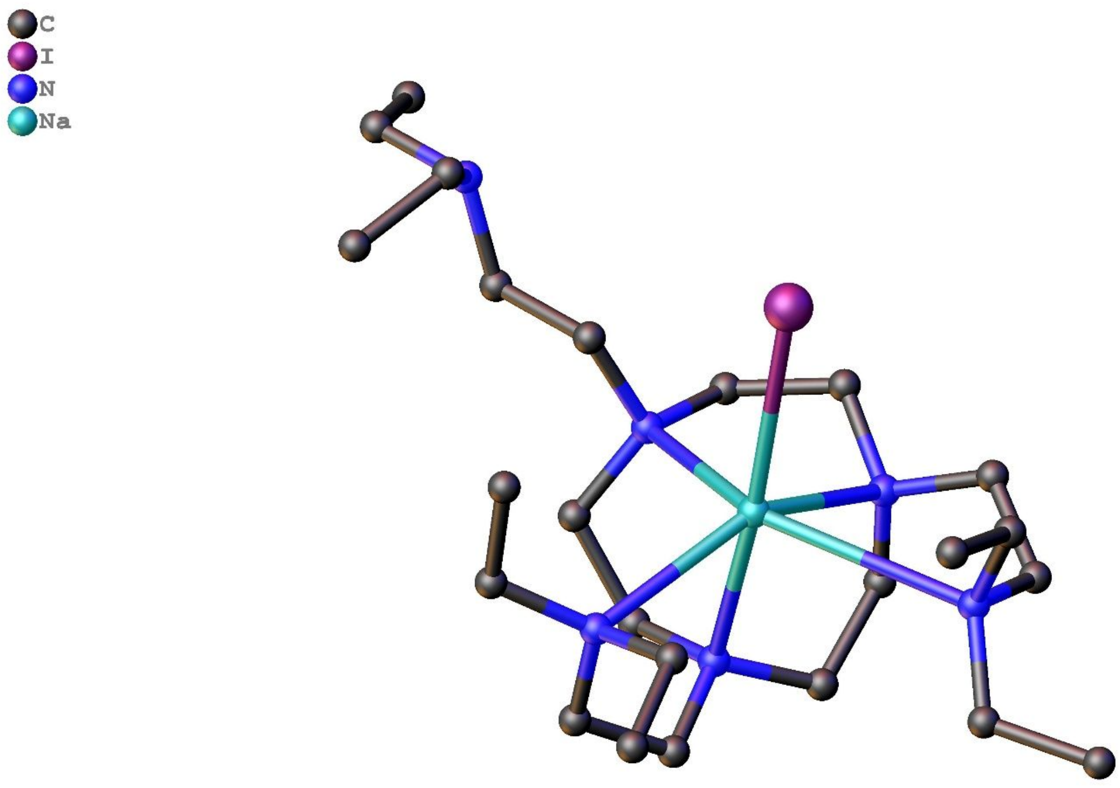

Figure S9. DFT Calculated optimized structure of **1**-Na.

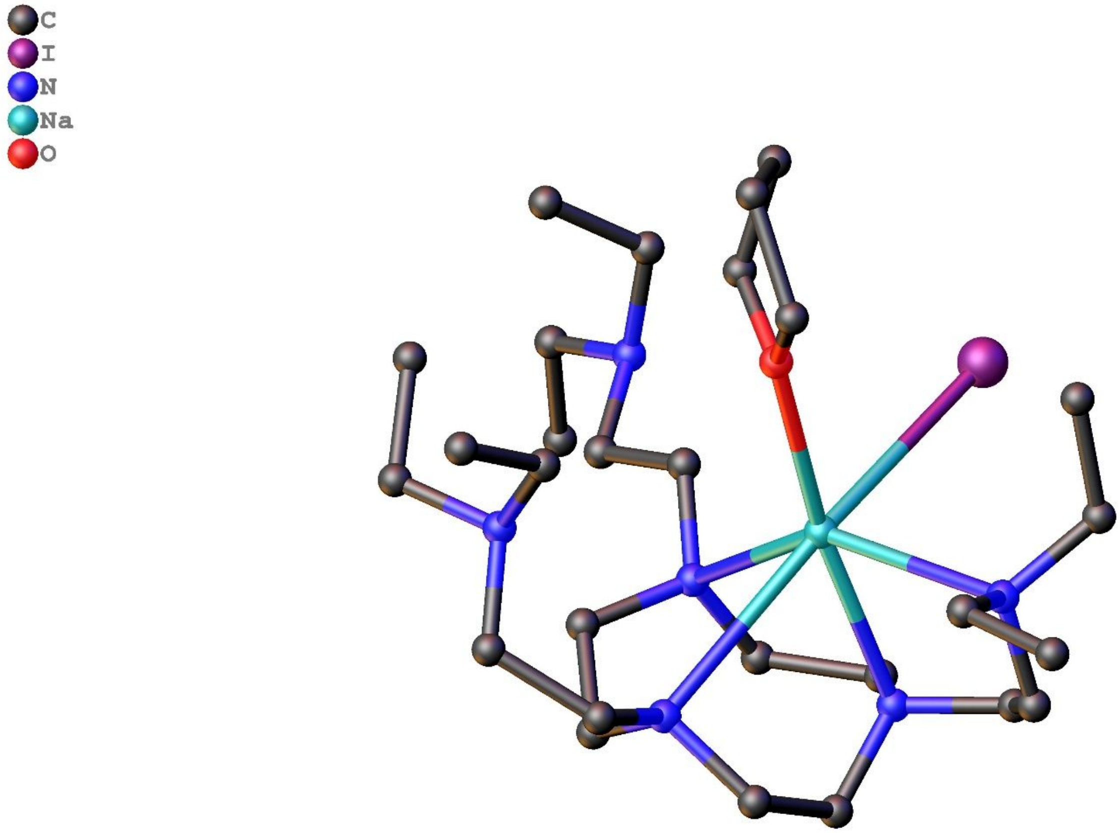

Figure S10. DFT Calculated optimized structure of **1**-Na-THF.

References:

[1] N. Davison, E. Falbo, P. G. Waddell, T. J. Penfold, E. Lu, A monomeric methyllithium complex: synthesis and structure. *Chem. Commun.* **2021**, *57*, 6205-6208.

[2] N. Davison, K. Zhou, P. G. Waddell, C. Wills, C. Dixon, S. –X. Hu, E. Lu, Versatile Coordination Modes of Multidentate Neutral Amine Ligands with Group 1 Metal Cations. *Inorg. Chem.* **2022**, *61*, 3674-3682.

[3] CrysAlisPro, Rigaku Oxford Diffraction, Tokyo, Japan.

[4] Sheldrick, G.M. *Acta Crystallogr., Sect. A: Found. Crystallogr.* **2015**, *71*, 3-8.

[5] Sheldrick, G.M. *Acta Crystallogr., Sect. A: Found. Crystallogr.* **2008**, *64*, 112-122.

[6] Dolomanov, O.V.; Bourhis, L.J.; Gildea, R.J.; Howard, J.A.K.; Puschmann, H. *J. Appl. Cryst.* **2009**, *42*, 339-341.

[7] VandeVondele J, Krack M, Mohamed F, Parrinello M, Chassaing T, Hutter J. Quickstep: Fast and accurate density functional calculations using a mixed Gaussian and plane waves approach. *Computer Physics Communications*. **2005** Apr 15;167(2):103-28.

[8] Mardirossian N, Head-Gordon M. ωB97X-V: A 10-parameter, range-separated hybrid, generalized gradient approximation density functional with nonlocal correlation, designed by a survival-of-the-fittest strategy. *Phys. Chem. Chem. Phys.* **2014**, *16*, 9904.

[9] VandeVondele J, Hutter J. Gaussian basis sets for accurate calculations on molecular systems in gas and condensed phases. *J. Chem. Phys.* **2007**, *127*, 114105.

[10] Gœedecker S, Teter M, Hutter J. Separable dual-space Gaussian pseudopotentials. *Phys. Rev. B* **1996**, *54*, 1703.

[11] Krack M. Pseudopotentials for H to Kr optimized for gradient-corrected exchange-correlation functionals. *Theo. Chem. Acc.* **2005**, *114*, 145-152.

[12] Hartwigsen C, Gœdecker S, Hutter J. Relativistic separable dual-space Gaussian pseudopotentials from H to Rn. *Phys. Rev. B* **1998**, *58*, 3641.

[13] Guidon M, Hutter J, VandeVondele J. Robust periodic Hartree−Fock exchange for large-scale simulations using Gaussian basis sets. *J. Chem. Theo. Comp.* **2009**, *5*, 3010.

[14] Guidon M, Hutter J, VandeVondele J. Auxiliary density matrix methods for Hartree−Fock exchange calculations. *J. Chem. Theo. Comp.* **2010**, *6*, 2348.
